# Sub-Hz fundamental, sub-kHz integral linewidth self-injection locked 780 nm hybrid integrated laser

**DOI:** 10.1038/s41598-024-76699-x

**Published:** 2024-11-18

**Authors:** Andrei Isichenko, Andrew S. Hunter, Debapam Bose, Nitesh Chauhan, Meiting Song, Kaikai Liu, Mark W. Harrington, Daniel J. Blumenthal

**Affiliations:** 1 Department of Electrical and Computer Engineering, University of California Santa Barbara, Santa Barbara, CA 93106 USA; 2 Present Address, Time and Frequency Division, National Institute of Standards and Technology, Boulder, CO 80305 USA

**Keywords:** Photonic integration, Laser stabilization, Rubidium, Narrow-linewidth lasers, Lasers, LEDs and light sources, Atom optics

## Abstract

Today’s precision experiments for timekeeping, inertial sensing, and fundamental science place strict requirements on the spectral distribution of laser frequency noise. Rubidium-based experiments utilize table-top 780 nm laser systems for high-performance clocks, gravity sensors, and quantum gates. Wafer-scale integration of these lasers is critical for enabling systems-on-chip. Despite progress towards chip-scale 780 nm ultra-narrow linewidth lasers, achieving sub-Hz fundamental linewidth and sub-kHz integral linewidth has remained elusive. Here we report a hybrid integrated 780 nm self-injection locked laser with 0.74 Hz fundamental and 864 Hz integral linewidths and thermorefractive-noise-limited 100 Hz^2^/Hz at 10 kHz. These linewidths are over an order of magnitude lower than previous photonic-integrated 780 nm implementations. The laser consists of a Fabry-Pérot diode edge-coupled to an on-chip splitter and a tunable 90 million Q resonator realized in the CMOS foundry-compatible silicon nitride platform. We achieve 2 mW output power, 36 dB side mode suppression ratio, and a 2.5 GHz mode-hop-free tuning range. To demonstrate the potential for quantum atomic applications, we analyze the laser noise influence on sensitivity limits for atomic clocks, quantum gates, and atom interferometer gravimeters. This technology can be translated to other atomic wavelengths, enabling compact, ultra-low noise lasers for quantum sensing, computing, and metrology.

**Supplementary Information:**

The online version contains supplementary material available at 10.1038/s41598-024-76699-x.

## Introduction

Integrated ultra-narrow linewidth visible and near-IR emission lasers are critical for the miniaturization, improved reliability, and scaling of atomic systems such as quantum computing^1^, precision sensing^2^, and timekeeping^3^. These applications have stringent requirements on the spectral noise properties of the laser over several decades of frequency offset from carrier. For systems that involve the manipulation and interrogation of atoms and qubits, evaluating the probing laser frequency noise requires consideration of hyperfine atomic transitions and sidebands as well as Fourier frequency components generated by pulse sequencing and other control signals applied to the laser and atom. For example, ultra-narrow linewidth lasers at rubidium atom wavelengths such as 780 nm are key for applications including two-photon atomic clocks^4^, cold atom interferometer sensors^2^, and neutral atom quantum computing^1^. Traditionally, these systems utilize costly external cavity lasers^5^, bulky table-top reference cavities, and frequency doubled C-band lasers^4^. Photonic integration using wafer-scale CMOS foundry compatible platforms is critical to realizing compact, lightweight, low cost and portable atomic and quantum systems and enables further integration with passive and active photonic components. Visible and near-IR (NIR) lasers, and in particular 780 nm, that achieve sub-Hz fundamental and sub-kHz integral linewidth regimes and deliver moderate output power and frequency agility are of interest for these applications. To date, this level of performance at 780 nm in a wafer-scale platform has remained elusive. One approach is direct-drive (direct emission without frequency conversion) optical self-injection locking (SIL)^6^. Bulk-optic 780 nm SIL lasers have achieved Hz-level fundamental linewidths^7^. The next step in quantum and atomic systems is realization of direct-drive high performance visible and near-IR lasers in a photonic integrated, CMOS foundry-compatible platform. This milestone will impact a wide range of applications including portable atomic clocks^8^ and space-based quantum sensors^9^.

Realizing ultra-low laser frequency noise in this regime requires a high level of frequency-selective feedback commonly achieved with an ultra-high-quality factor (UHQ) optical cavity. Hertz-level linewidth 780 nm lasing has been achieved with SIL configurations that lock a single-frequency diode to a bulk optic crystalline UHQ whispering gallery mode resonator (WGMR) reaching a fundamental linewidth of 5 Hz and a 1/$\:\pi\:$ integral linewidth of 1.4 kHz^7^. However, WGMRs pose a challenge for more complex systems-on-chip integration, wafer-scale foundry processing, and low cost and size. Integration using CMOS foundry-compatible processes such as the ultra-low loss silicon nitride (SiN)^10^ is key to reducing cost and weight and improving robustness to environmental disturbances. Today, at 1550 nm, integrated high-Q SiN resonators have been used to realize ultra-low fundamental linewidths of less than 100 mHz^11,12^ and integral linewidths to 36 Hz^13^. Translating this level of performance to the visible and NIR wavelengths has been challenging due to the increased optical losses and the higher cost or limited availability of laser diodes, particularly single-frequency semiconductor lasers such as distributed Bragg reflector (DBR) and distributed feedback (DFB) lasers. At 780 nm, corresponding to the $\:{D}_{2}$ transition in rubidium, multi-frequency Fabry-Pérot laser diodes (FPLDs) have been used in SIL with frequency-selective resonators to achieve both single mode operation and linewidth narrowing. For example, FPLD SIL to free-space-coupled narrow band filters have demonstrated 60 kHz beat linewidth^14^. SIL with FPLDs coupled to integrated, tightly-confined SiN resonators was recently demonstrated at six visible and NIR wavelengths including 780 nm where devices with 80,000 Q yielded 700 Hz fundamental and 50 kHz integral linewidths^15^. Intra-cavity SIL frequency-doubled single-frequency lasers achieved 12 Hz fundamental linewidth but with limited 30 $\:\mu\:$W output power in fiber^16^. Recently a 780 nm heterogeneous GaAs laser butt-coupled to a 10 M intrinsic Q SiN ring was used to reach 92 Hz fundamental linewidth^17^. A similar experiment with a 780 nm DFB and a 5 million Q resonator achieved 105 Hz^18^ and calculated that in resonators with 50 million Q the achievable fundamental linewidth could reach 3 Hz. To lower the noise and linewidth further, losses at 780 nm must be significantly lowered and the resonator Q increased. To date, the lowest reported loss at 780 nm is 0.1 dB/m in a silica wedge resonator^19^ and 0.2 dB/m in a germano-silicate platform^20^. The lowest reported visible losses and highest Q for fully integrated SiN resonators are 0.65 dB/m loss and 90 million intrinsic Q at 674 nm^21^, 0.36 dB/m and 145 million intrinsic Q at 780 nm^22^. Low linewidth integrated visible light lasers include a 674 nm stimulated Brillouin laser achieving a 12 Hz fundamental linewidth^23,24^. To reach the ultra-low frequency noise regime at 780 nm, new direct-drive laser technologies based on ultra-low loss waveguides and ultra-high Q, large mode volume resonators are needed.

Here we report demonstration of an advanced direct-drive atomic precision light source by realizing a sub-Hz fundamental, sub-kHz integral linewidth 780 nm laser for rubidium atom applications in a CMOS-foundry compatible, wafer-scale, silicon nitride integration platform. The high Q enables SIL of a low-cost FP laser diode, reducing the free running multi-frequency operation to a single mode laser with 864 Hz integral linewidth and fundamental linewidth 0.74 Hz (white frequency noise 0.24 Hz^2^/Hz). The laser consists of a single-bus coupled ring resonator, an intra-cavity power splitter, and an integrated tunable phase section. This design has multiple advantages over dual-bus resonator approaches^11,15,16^ such as a higher resonator loaded Q and the possibility to independently control output power and resonator coupling^25^ for SIL optimization^26^. The strong optical feedback enables extracting a 2 mW fiber-coupled output power using the intra-cavity splitter. This configuration and level of performance is possible due to the 0.57 dB/m waveguide loss, 90 million intrinsic resonator Q with a critically-coupled 20 dB extinction ratio and a large cavity mode volume providing a large number of intra-cavity photons and low thermorefractive noise (TRN) limit. We also demonstrate frequency tuning ability with temperature tuning of the output over 781 to 797 nm and a 2.5 GHz mode-hop-free-tuning by controlling integrated thermal tuners. We achieve performance comparable to, and at certain frequencies lower than, crystalline WGM bulk-optic resonator implementations^7^. In our demonstration the fundamental linewidth is over two orders of magnitude lower than that of direct-emission hybrid-integrated SIL^17,18^ and the integral linewidth (1/$\:\pi\:\:$integration) is over an order of magnitude lower than any chip-based 780 nm SIL laser, to the best of our knowledge. We investigate how the ultra-low laser frequency noise can improve the performance of three different Rb atom quantum technologies: lower short-term instability in a two-photon clock, enhanced acceleration sensitivity in a cold atom interferometer gravimeter, and one-photon quantum gates with errors below 10^− 4^. The platform performance is scalable to other visible atomic wavelengths^27^ where FP laser diodes are commercially available, opening the door to a wide variety of transitions across many atomic species such as Sr and Cs. These results represent orders of magnitude improvement in fundamental and integral linewidths over previous 780 nm integrated SIL demonstrations. The CMOS foundry platform and process are fully compatible with other passive and active components^10,28^, showing promise for full system-on-chip integration.

## Results

**Fig. 1:**
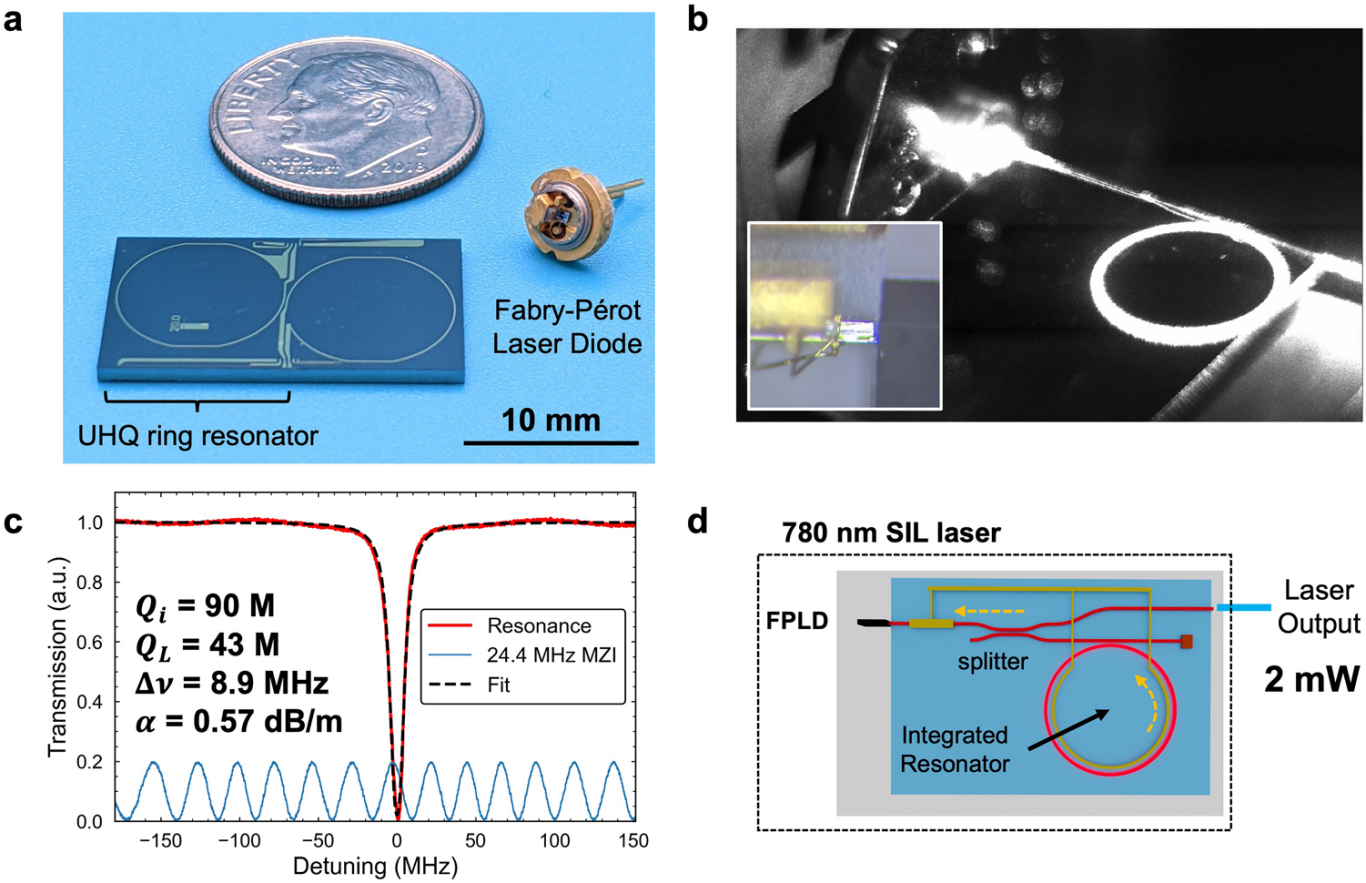
Hybrid-integrated chip-scale 780 nm self-injection locked laser. (**a**) Picture of the integrated ultra-high-Q (UHQ) ring resonator and Fabry-Pérot laser diode (FPLD) with a dime for scale. (**b**) Laser locked to a ring on resonance. Inset: edge coupling of the rotated FPLD. (**c**) Transmission spectrum of the resonator indicating a loaded quality factor ($\:{Q}_{L}$) of 43 million, intrinsic quality factor ($\:{Q}_{i}$​) of 90 million, loss $\:\alpha\:$ 0.57 dB/m, and a total linewidth of 8.9 MHz. The frequency tuning is calibrated with an unbalanced Mach-Zehnder interferometer (MZI, blue trace). (**d**) Schematic of the self-injection locked (SIL) laser comprising an edge-coupled FPLD to a chip with a splitter and resonator with thermal tuners for controlling the phase between the laser and resonator and the resonance detuning. The laser output is collected in an edge-coupled cleaved fiber.

The hybrid-integrated ultra-low frequency noise SIL consists of a commercial 780 nm FPLD in a TO-can package hybrid edge-coupled to an integrated ultra-high Q Si_3_N_4_ chip (Fig. 1a, b). The combination of nearly critical coupling, yielding a 20 dB extinction ratio, and 90 million intrinsic Q enables a strong frequency-selective back-reflection to the laser through resonant Rayleigh backscattering inside the resonator cavity^7^. The resonator, power splitter, and phase tuner are fabricated using the silicon nitride CMOS foundry-compatible process^10^ (Supplementary Note 3). The TM mode waveguide design is used due to its lower loss^29^. The FPLD is rotated 90 degrees to couple to the waveguide TM mode (Fig. 1b). The resonator intrinsic Q_i_ = 90 M and loaded Q_L_ =43 M are measured using an unbalanced Mach-Zehnder interferometer (MZI)^30^ resulting in a linewidth 8.9 MHz and a loss of 0.57 dB/m (Fig. 1c). The ring resonator radius is 5 mm corresponding to a 6.43 GHz free-spectral range (FSR). The laser diode is a commercial TO-can packaged laser (Thorlabs L785P090) with the lid removed and edge-coupled directly to the chip. We demonstrate and compare a version with the alignment optimized with a multi-axis stage and a hybrid-packaged version with the laser bonded with respect to the chip.

The strength of the optical feedback from the resonator is sufficiently strong to allow for extracting the laser output with an intra-cavity directional coupler splitter achieving 2 mW output power (Fig. 1d), over an order of magnitude greater than that of the lowest fundamental linewidth integrated chip-scale SIL^16^. The waveguide tapers down from 4 μm width in the resonator to 2 μm width at the edge for increased coupling to the laser and output fiber. The measured coupling between the rotated FPLD and the chip is ~ 4 dB.

**Fig. 2:**
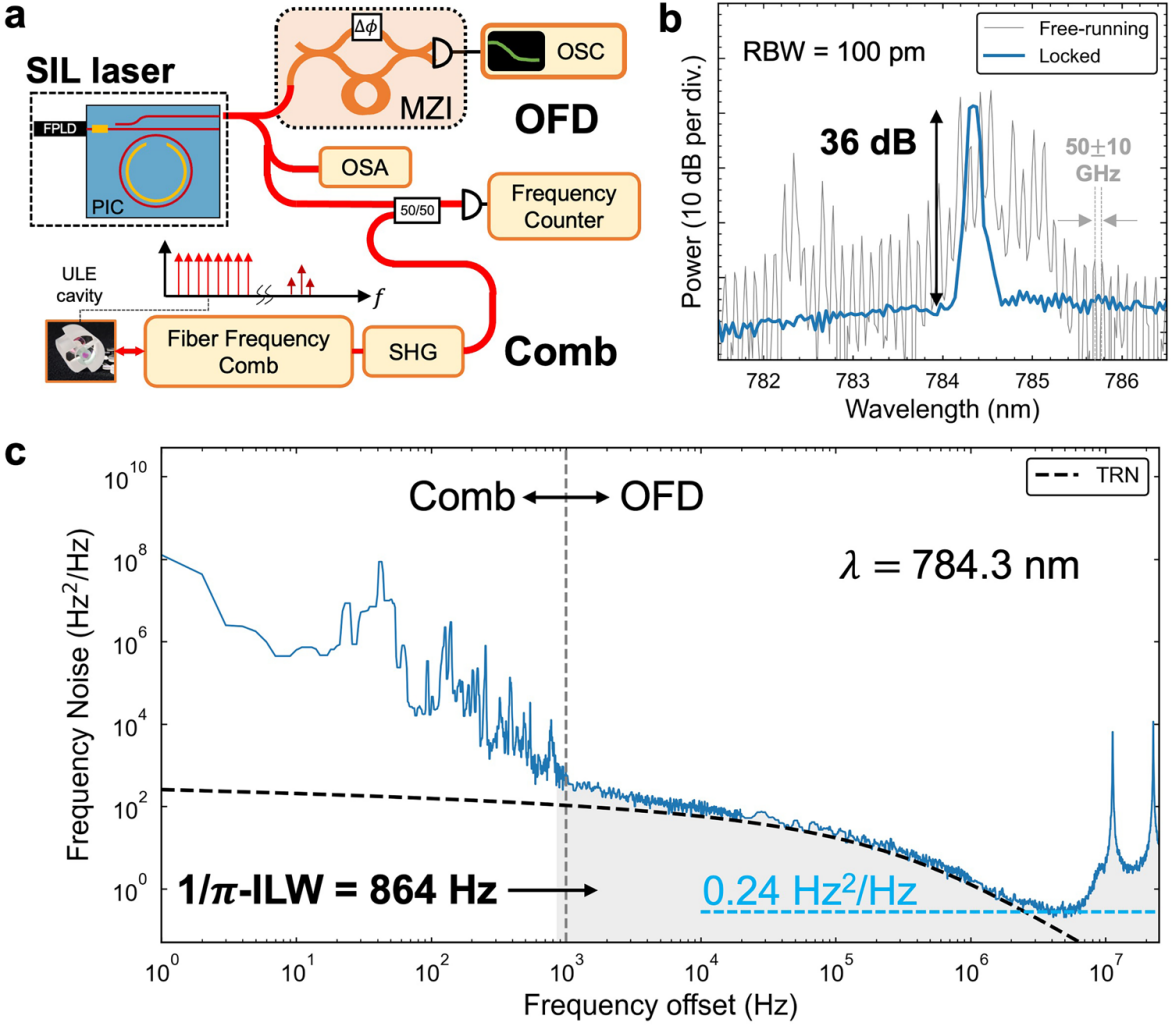
SIL laser characterization. (**a**) Laser frequency noise measurements with fiber MZI optical frequency discriminator (OFD) and second harmonic generation (SHG) frequency-doubled cavity-stabilized fiber frequency comb for beat-note measurements. (**b**) OSA free running vs. single mode with 36 dB side-mode-suppression ratio (SMSR). (B) Frequency noise for the SIL laser with data stitched between comb beat note and OFD data. Spurs at multiples of 11.3 MHz correspond to the free-spectral range of the OFD MZI and do not contribute to the integral linewidth calculation.

The SIL results in single mode operation with ultra-low frequency noise measured at frequency offsets spanning from 1 Hz to 25 MHz (Fig. 2). The frequency noise reaches a minimum value of 0.24 Hz^2^/Hz corresponding to a fundamental linewidth of 0.74 Hz and an 864 Hz 1/$\:\pi\:$ integral linewidth^13^ while operating at an in-fiber output power 0.84 mW. The details of the SIL laser characterization are shown in Fig. 2a. Single-mode narrow-linewidth lasing is verified by observing a strong lasing peak on the OSA (Fig. 2b) and monitoring the fringes of an unbalanced fiber MZI while ramping a fiber phase shifter in the fiber MZI. SIL results in a selection of an FPLD cavity mode and we observe a side-mode-suppression-ratio (SMSR) of over 36 dB at the output wavelength 784.3 nm. The frequency noise is measured with a combination of the fiber MZI optical frequency discriminator (OFD)^23^ and a heterodyne beat-note with cavity-stabilized fiber frequency comb (Fig. 2a). The frequency comb is self-referenced and the optical refence frequency is locked to a Hz-level ULE-cavity-stabilized C-band laser. The stabilized comb light is frequency doubled to 780 nm, combined with the 780 SIL laser using a 50:50 fiber coupler, and detected on a photodetector. The beat-note signal is electronically filtered to isolate an individual comb line and recorded on a frequency counter. The frequency noise from the two independent measurement techniques is stitched together (Fig. 2c) and the details are described in Supplementary Note 2 and Supplementary Fig. S1. Importantly, the frequency noise reaches the simulated resonator TRN limit^31^ which is low due to the large optical mode volume^13^ of the 5 mm radius, 6.43 GHz FSR device. We estimate the TRN-limited performance to yield a 320 Hz $\:1/\pi\:$ integral linewidth.

**Fig. 3:**
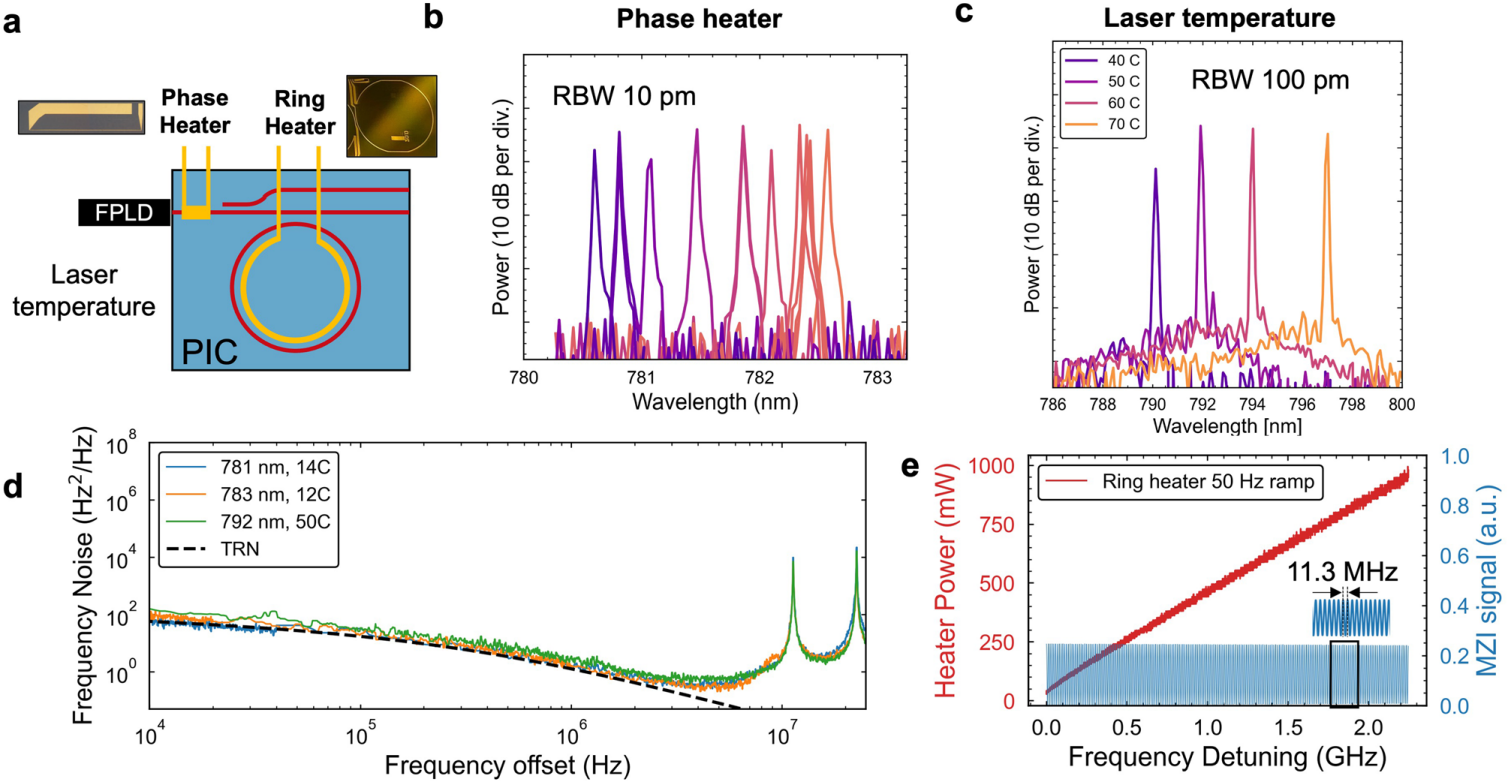
SIL laser tuning and multi-wavelength operation. (**a**) Schematic for the control knobs used in the tuning. Insets: micrographs of metal thermal tuners for phase and ring. (**b**) SIL at different wavelengths achieved by selecting a different laser mode. (**c**) Broad wavelength tuning by changing the laser diode temperature. (**d**) Frequency noise of the device at different wavelengths. (**e**) Mode-hop-free tuning across over 2 GHz by ramping the ring heater at 50 Hz.

Next, we demonstrate laser wavelength and frequency tuning and agility using metal heaters for controlling the phase between the laser and the resonator and the ring resonance detuning (Fig. 3a). We use a straight heater on the input waveguide near the laser to control the SIL phase and to achieve single-mode operation at several wavelengths corresponding to where the resonator and FPLD modes are well overlapped. We achieve single-mode lasing in the wavelength range 780.6 to 782.6 nm with > 30 dB SMSR for a constant FPLD current and fixed FPLD and PIC temperature, representing a coarse tuning ability (Fig. 3b). For laser operation over a broader wavelength range, the FPLD gain spectrum can be tuned by changing the laser temperature with a tuning strength of 0.2 nm/K. For the packaged laser, we demonstrate single mode lasing at wavelengths 790 to 796 nm at an elevated FPLD temperature (Fig. 3c) and measured the frequency noise of the laser operating at 792 nm, resulting in a similar noise performance (Fig. 3d). Despite the larger FPLD to chip coupling loss, the packaged laser achieves a 1.6 Hz FLW and 3.1 kHz $\:1/\pi\:\:$ILW and 0.7 mW output power (Supplementary Note 4, Supplementary Fig. S4). Future packaging efforts will focus on reduced coupling loss and an ability to operate at a range of FPLD and chip temperature to access absolute wavelengths of interest.

The ring heater is used to finely control the resonance position to tune the laser frequency. The resonance tuning strength is measured to be 26 MHz/mW during quality factor measurements using a single-frequency DBR laser (Supplementary Fig. S2, Supplementary Note 3). During SIL the FPLD lasing mode closely tracks the cavity resonance. For a stable single-mode SIL we apply a voltage ramp to the ring heater at a 50 Hz rate and measure the laser frequency detuning using a fiber MZI (FSR = 11.3 MHz). Figure 3(e) shows a triggered trace of the ramp and the continuous MZI fringes demonstrating a mode-hop-free tuning range of at least 2.5 GHz. The sweep range is sufficient to cover a hyperfine family in rubidium saturation absorption spectroscopy which requires at least several 100 MHz to identify a particular hyperfine peak. The relatively large heater power (100s mW) required for this sweep can be related to the thermal response time of the heater and the sweep rate. In the future, integrated of PZT-on-SiN^28^ can be used to significantly lower the power requirement as well as improve modulation bandwidth for SIL laser frequency detuning and stabilization to spectroscopy.

## Discussion

We demonstrate a significant advance in the development of chip-scale ultra-narrow linewidth self-injection locked lasers at 780 nm. We achieve a 0.74 Hz fundamental linewidth and a 864 Hz integral linewidth with a frequency noise that closely follows the thermo-refractive-noise limit from 10 kHz to 5 MHz. The laser is realized with a multi-longitudinal-mode Fabry-Pérot laser diode directly edge-coupled to the SiN chip. The strong SIL feedback provided by the 90 million Q resonator cavity promotes single-longitudinal-mode lasing with 36 dB SMSR. The laser output is extracted using an on-chip directional coupler power splitter and delivers a maximum of 2 mW of optical power in the cleaved collection fiber. The metallized thermally tunable resonator cavity provides precision frequency tuning of the locked laser, achieving a 2.5 GHz mode-hop-free tuning range.

### Comparison to other work and lasers

**Fig. 4:**
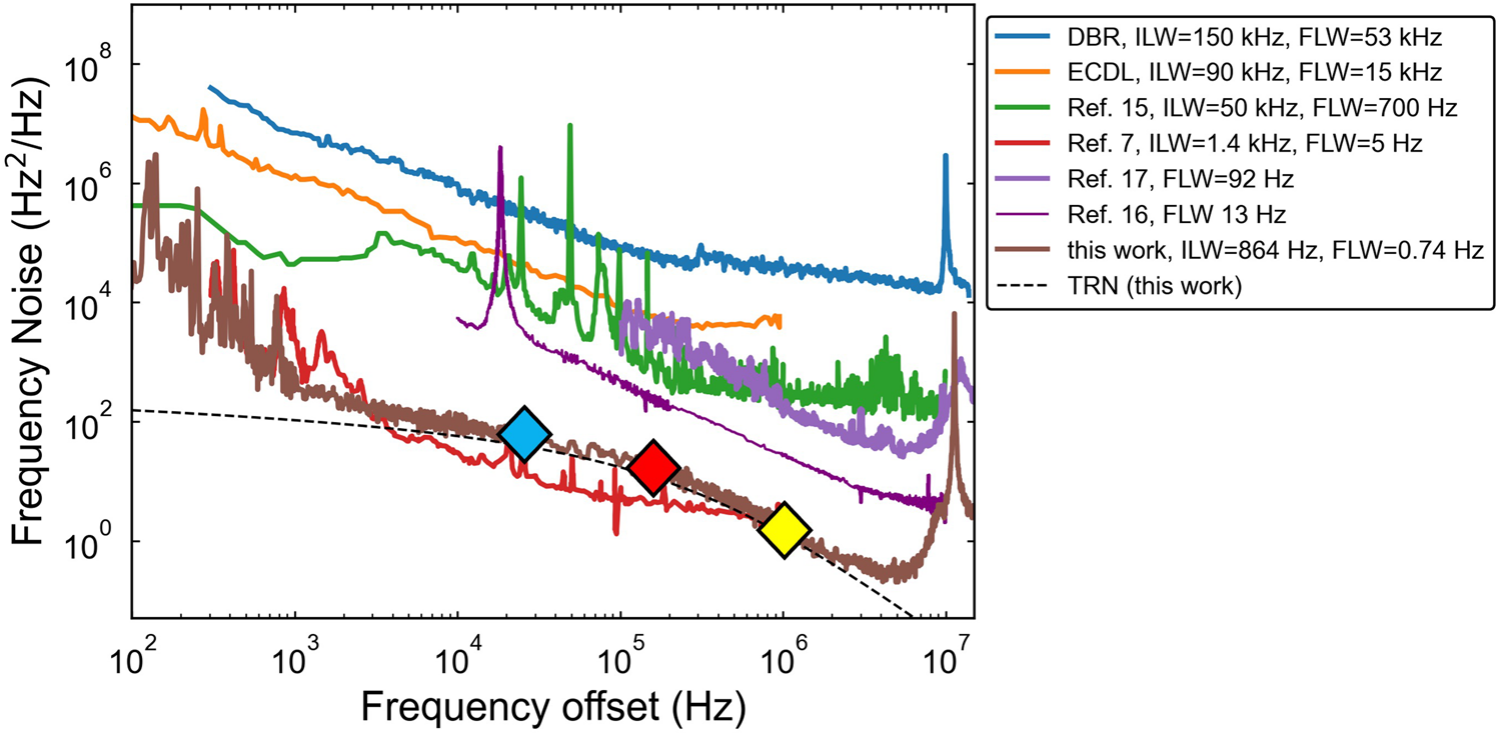
Laser frequency noise comparison. Non-integrated distributed Bragg reflector (DBR) and external cavity diode laser (ECDL) from^32^. Integral linewidths (ILW) listed in the legend is calculated $\:1/\pi\:$ reverse integration method, when applicable. FLW: fundamental linewidth. Diamonds corresponds to relevant frequency offsets discussed in Table 1: 24 kHz (blue), 160 kHz (red), 1 MHz (yellow).

 As shown in Fig. 4, we achieve a fundamental linewidth of 0.74 Hz compared to 5 Hz in a previous WGMR bulk-optic demonstration^7^, over two orders of magnitude lower than that of 780 nm direct-emission SIL to integrated resonators^15,17^, and frequency noise at certain frequency offset that is over 3 orders of magnitude lower than in recently reported hybrid-integrated SIL^15,17^ (Fig. 4). Our fundamental linewidth is below that predicted by a laser-dynamics theoretical model for DFB-coupled SIL to a resonator with 50 million Q which states an achievable 3 Hz fundamental linewidth^18^. We provide an estimate for the minimum achievable fundamental linewidth in Supplementary Note 2. The FPLD SIL is much lower frequency noise than DBR and ECDL^32^ demonstrations commonly used in rubidium-related experiments such as two-photon optical clocks at 778 nm. The FPLD can support a broad wavelength range and we show operation near the Rb $\:{D}_{1}$ and $\:{D}_{2}$ wavelengths, limited by the temperature control of the cavity and mode selection for optimal feedback. Single-frequency lasers such as DFBs have been used extensively for chip-based SIL demonstrations in the C-band^16^. Using the FPLD is a low-cost alternative to a single-frequency diode when iterating packaging development because direct edge-coupling exposes the laser facet to potential damage. On the other hand, using a FPLD poses a challenge because SIL with a low TRN cavity (and low FSR) necessitates that there will be several resonator modes within the FPLD gain bandwidth. This can lead to mode competition, locking instability, or multi-mode lasing and limits the locked tuning range compared to SIL with much larger FSR resonators. Addressing this would require careful mode mapping to reliably reach absolute wavelengths^33^ as different resonator modes have different levels of resonant Rayleigh back-reflection. We estimate the back-reflection for different resonances in Supplementary Note 3 and Supplementary Fig. S3. For low-TRN coil resonators with sub-GHz FSR^11,13^, it is possible that stable SIL can only be achieved with single-frequency DFB or DBR laser diodes.

**Table 1:** Atomic and quantum laser frequency noise requirements and performance limits and comparison between this work and free-running semiconductor lasers.

Application	Offset frequency	Frequency noise at offset	Noise source^*^	Laser frequency noise limited performance	
		This work		DBR^**^/ ECDL^**^/ SIL^15^	This work
Cold atom interferometer gravimeter^39^	24 kHz, blue diamond in Fig. 4	55 Hz^2^/Hz	Gravity sensitivity limited by Raman laser frequency noise^***^ $\:{\sigma\:}_{\varphi\:}^{2}={\int\:}_{{f}_{x}}^{\infty\:}{H\left(f\right)}^{2}{S}_{{\Delta\:}\nu\:}\left(f\right)df$ $\:{\sigma\:}_{g}=\frac{{\sigma\:}_{\varphi\:}}{{g\:k}_{\text{e}\text{f}\text{f}\:}{T}^{2}\sqrt{{\Delta\:}f}}$	124 / 53 / 44 nm/s^2^/$\:\sqrt{\text{H}\text{z}}$	4 nm/s^2^/$\:\sqrt{\text{H}\text{z}}$
2-photon atomic wavelength reference at $\:{f}_{0}$ for 778 nm laser ^4^	160 kHz, red diamond in Fig. 4	15 Hz^2^/Hz	Short-term ($\:\tau\:\approx\:1$ s) instability due to intermodulation (IM) effect at $\:{f}_{m}$modulation frequency^32,35^ $\:{\sigma\:}_{y}^{\left(IM\right)}\left(\tau\:\right)=\:\frac{{\left[{S}_{{\Delta\:}\nu\:}\left(2{f}_{m}\right)/{f}_{0}^{2}\right]}^{1/2}}{2\:\sqrt{\tau\:}}$	$\:1\times\:{10}^{-12}\:/\:9\times\:{10}^{-14}\:/$ $\:4\times\:{10}^{-14}$	$\:5\times\:{10}^{-15}$
High-fidelity neutral-atom qubit operations^36^	1 MHz, yellow diamond in Fig. 4	1.5 Hz^2^/Hz	Single-photon gate operation averaged error for Rabi frequency $\:{{\Omega\:}}_{0}/2\pi\:=\:$1 MHz and a $\:\pi\:$ pulse^37^. $\:\stackrel{-}{\mathcal{E}}=\frac{8\:{\pi\:}^{2}}{3\:}{\int\:}_{{f}_{x}}^{\infty\:}{S}_{{\Delta\:}\nu\:}\left(f\right)\:H\left(f\right)\:df$	$\:2\times\:{10}^{-1}\:/\:2\times\:{10}^{-2}\:/$ $\:3\times\:{10}^{-2}$	$\:2\times\:{10}^{-5}$

^*^ Noise contribution related to laser frequency noise, assuming all other noise sources are not considered in evaluating the system performance. Calculations with integration assume $\:{f}_{x}$ = 100 Hz for the gravimeter and $\:{f}_{x}$ = 1 kHz for qubit operations.

^**^ Free-running lasers such as a distributed Bragg reflector (DBR) semiconductor laser and an external cavity diode laser (ECDL). Data for the ECDL is taken from^32^.

^***^ Using cold atom interferometer parameters from^38^. $\:g\:$is the acceleration due to gravity (9.8 m s^-^^2^, $\:{k}_{\text{e}\text{f}\text{f}\:}$is the effective wave vector of the two Raman probe lasers, $\:T\:$is the time interval between Raman pulses, and $\:{\Delta\:}f\:\:$is the atom interferometer cycle rate or bandwidth.

### Analysis of the requirements for atomic experiments

 The development of integrated, narrow-linewidth laser sources is critical for atomic and quantum systems and requires careful consideration of the frequency noise requirements associated with various applications. We analyze the expected performance enhancement delivered by this frequency noise spectrum to three applications: (1) two-photon optical clock, (2) a high-fidelity quantum computing gate, and (3) an atom interferometer gravimeter. The performance of these quantum systems is influenced by the spectral distribution of the incident optical local oscillator (OLO) frequency noise which affects the transition probabilities between atomic energy levels. Understanding the impact of FN, rather than stating a total linewidth, is essential because applications have distinct sensitivity to noise at different frequency bands. Low frequency drift can cause the laser to deviate from the atomic transition absolute frequency. Mid-frequency (offset from carrier) noise can dominate the residual lock loop noise, and high frequency noise can lead to crosstalk with neighboring hyperfine transitions and motional sidebands. In systems that are periodically interrogated by the OLO, particularly with fast pulses, noise at high frequencies can alias into the readout of the atomic or quantum sensor^34^. To quantify the effect of laser frequency noise on overall performance, an application-specific calculation can weigh the importance of frequency components in the laser frequency noise distribution. We summarize how the measured FN can be used to estimate a laser-related performance limit in three distinct rubidium atomic applications that could benefit from photonic integration for future scalability and portability (Table 1). The methods used to develop Table 1 are applicable to many other wavelengths, transitions, and applications. We compare the performance of our laser to that of a typical free-running semiconductor Distributed Bragg Reflector (DBR) laser, an external cavity diode laser (ECDL), and an integrated SIL result^15^ and note relevant frequency offsets. Details of these calculations are provided in Supplementary Note 5.

### Two-photon optical atomic clocks

The process of optically interrogating an atomic transition introduces a time-varying sensitivity to the optical local oscillator laser frequency noise^34^. In the case of atomic frequency standards such as a two-photon rubidium optical clocks, this effect is known as intermodulation noise^35^. The short-term clock instability scales with the optical local oscillator laser frequency noise at twice the modulation frequency $\:{f}_{m}$ used in the stabilization^32^. We calculate that our reported ultra-low frequency noise SIL laser, if used as the 778 nm probe can achieve a short-term ($\:\tau\:\approx\:$ 1 s) stability of $\:5\times\:{10}^{-15}$ at 1 s, almost 18x lower than the calculated limit in a previously reported laser-limited compact optical standard^32^. We note that this is a specific case when intermodulation noise is the dominant limiting factor; in practice the short-term stability is usually limited by shot noise associated with fluorescence signal detection.

### High fidelity quantum computing gates

 For neutral atom quantum computing, excess laser frequency noise can limit gate fidelities due to Rabi oscillation dephasing^36,37^. When the qubit-driving laser is actively stabilized to a bulk reference cavity, frequency noise servo bumps from the finite lock bandwidth can impact gate fidelity. The error contribution is largest for frequency noise near the Rabi frequency, which can limit the choice of Rabi frequency and gate protocols. Filtering this noise with a table-scale high-finesse cavity has been shown to increase Rydberg state coherence times from 7 $\:\mu\:$s to over 20 $\:\mu\:$s^36^. Gates based on one-photon, two-photon, and entangling Rydberg transitions have increasingly stringent laser frequency noise requirements, with estimates of white frequency noise below 100 Hz^2^/Hz, 20 Hz^2^/Hz, and 5 Hz^2^/Hz, respectively (for Rabi frequency 1 MHz and 10^− 4^ gate error)^37^. Table 1 shows a relation between driving laser frequency noise and averaged quantum gate errors incurred during one-photon gate operations for lasers operating near 780 nm.

### Gravimeters

 A similar pulsed interrogation scheme can cause sensitivity limits in cold atom interferometer gravimeters used for gravity surveys^2,38^. The probe Raman laser frequency noise affects gravity measurement sensitivity through a transfer function $\:H\left(f\right)$ with parameters related to the cold atom experiment such as sensor bandwidth and Raman laser pulse duration^38^. We calculate the gravity measurement sensitivity limit solely due to the Raman laser frequency noise. In general, gravimeter measurements have required lower-noise, bulky ECDL lasers because free-running semiconductor lasers may introduce noise contributions greater that introduced by vibrations and degrade the gravimeter sensitivity^38^.

The integration of foundry-compatible photonics offers significant potential to enable fully integrated atomic and quantum systems at the chip scale, improving scalability and robustness. Our ultra-low-loss platform that enables these record-low frequency noise results has also been demonstrated in other relevant chip-scale functionalities. Importantly, the requirements of these applications extend beyond achieving low frequency noise; precise control over the absolute laser frequency is also essential. In this regard, our ultra-high Q resonator can be metallized with a thermal tuner such that the ultra-low optical loss is maintained, enabling tuning a locked laser for stabilization to atomic spectroscopy^39^. Advancements such as faster modulation with PZT-on-SiN^28^ have also been demonstrated without affecting optical loss, presenting opportunities for pulse generation and implementing stabilization loops for atomic systems-on-chip. Furthermore, SiN photonic integrated beam delivery in a cold atom magneto-optical trap has achieved large output beam diameters, enabling successful trapping of over a million rubidium atoms^40^. This atom number is promising for surpassing shot noise limits in sensors such as atom interferometers. These results demonstrate the potential of integrating an ultra-low linewidth SIL laser as a probe for compact atomic sensors, clocks, and a wide range of precision atomic and quantum technologies.

## Electronic supplementary material

Below is the link to the electronic supplementary material.

Supplementary Material 1

## Data Availability

The datasets generated during and/or analysed during the current study are available from the corresponding author on reasonable request.
