# Supplementary material for: Sub-Hz fundamental, sub-kHz integral linewidth self-injection locked 780 nm hybrid integrated laser
Source: Sci Rep. 2024 Nov 18;14:27015. doi: 10.1038/s41598-024-76699-x (PMC11574317; doi:10.1038/s41598-024-76699-x)
Supplement: Supplementary file 1 — Supplementary Material 1 [file 41598_2024_76699_MOESM1_ESM.pdf]

Supplementary Information

Sub-Hz Fundamental, Sub-kHz Integral Linewidth Self-Injection Locked 780 nm Hybrid Integrated Laser

Andrei Isichenko[1], Andrew S. Hunter[1], Debapam Bose[1], Nitesh Chauhan[1,2], Meiting Song[1], Kaikai Liu[1], Mark W. Harrington[1], and Daniel J. Blumenthal[1*]

[1] Department of Electrical and Computer Engineering, University of California Santa Barbara, Santa Barbara, CA 93106 USA

[2] Present Address, Time and Frequency Division, National Institute of Standards and Technology, Boulder, CO, USA

* Corresponding author (danb@ucsb.edu)

Supplementary Note 1: Introduction

In this Supplementary Information, we discuss further details with respect to the laser noise characterization and resonator design and characterization, laser packaging, and calculations for atomic and quantum systems.

Supplementary Note 2: Laser noise measurements

We characterize the laser frequency noise and relative intensity noise as shown in Supplementary Fig. S1. The full frequency noise from 1 Hz to 20 MHz is determined by stitching the data from two independent measurement techniques: the optical fiber MZI optical frequency discriminator (OFD) and comb beat note. The schematic of this measurement is shown in Figure 2a of the main text. We measured our OFD measurement noise floor, which has contributions from the balanced photodetector (BPD) noise and the digital oscilloscope digitization noise. The oscilloscope noise is minimized by decreasing the voltage sampling window to improve the digital resolution. We use the Thorlabs PDB415A detector and the noise is estimated by measuring the power spectral density (PSD) of the detector voltage signal measured with no optical power and converted to an equivalent frequency noise spectrum (in $Hz^2\,Hz^{-1}$) using the equation[1,2]:

$$S_{\mathrm{PD,equiv}}(f) = S_{\mathrm{PD}}(f)\left(\frac{f}{\sin(\pi f \tau_D) V_{pp,\mathrm{OFD}}}\right)^2 \qquad (1)$$

where τ_D is the MZI optical delay and $V_{pp,\mathrm{OFD}}$ is the peak-to-peak voltage of the measured MZI fringes during the OFD measurement of the SIL laser. The other noise sources in the frequency

noise measurement relate to the integrated resonator cavity. The photothermal noise (PTN) and thermo-refractive noise (TRN) contributed to the thermally driven frequency instability within the resonator[3]. The TRN is due to the fundamental thermodynamic fluctuations of solids and the PTN arises from the thermal fluctuations driven by power absorbed from a fluctuating optical field [4,5]. Our SIL laser frequency noise follows the estimated TRN limit at frequency offsets between 3 kHz and 3 MHz. Below 3 kHz, the high intra-cavity optical power contributes optical fluctuations and therefore the PTN. The inputs to our PTN model include the estimated on-chip optical power coupled to the resonator, the fraction of optical power absorbed in the cavity, the photothermal absorption resonance redshift, and the SIL output relative intensity noise (RIN) measured with a photodetector (Fig. S1b).

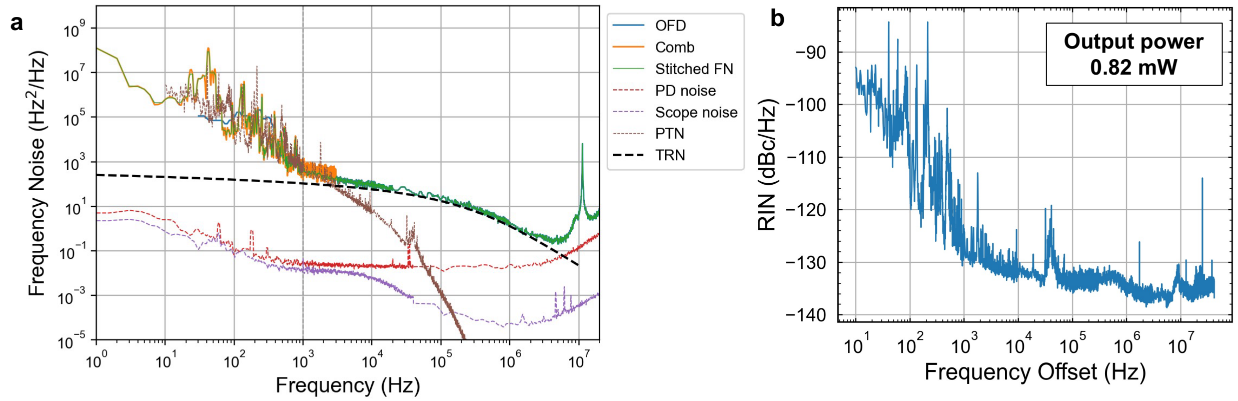

Supplementary Fig. S1. Laser noise and noise floors. a) SIL laser frequency noise measured with the fiber MZI optical frequency discriminator (OFD) and a beat-note with cavity-stability, frequency-doubled fiber comb (Comb). The photodetector (PD) and scope noise floors are relevant for the OFD noise. The photothermal noise (PTN) is calculated from the relative intensity noise (RIN) and the thermorefractive noise (TRN) is calculated from device geometry and a thermodynamical model[4]. b) RIN measurement for the SIL laser for a laser output power of 0.82 mW at 40 mA laser current. The shot noise limit is -152 dBc/Hz.

Estimated minimum achievable fundamental linewidth. We estimate the achievable fundamental linewidth (FLW) based on analysis for FLW reduction used in previous work with single-frequency[6,7] and FP lasers[8]. The fundamental linewidth (FLW) reduction given by:

$$\Delta\nu_{FLW} = \Delta\nu_{FLW_0}\left(\frac{Q_d}{Q_L}\right)^2\left(\frac{1}{16\,C^2\Gamma_m^2(1+\alpha_H^2)}\right) \quad (2)$$

where Q_d and Q_L are the laser and PIC resonator quality factors, C is the power coupling efficiency between the laser and PIC, and α_H is the linewidth enhancement factor of the laser. To estimate the FLW reduction in a multi-frequency FPLD, the SIL can be treated as a two-step process with mode selection and linewidth narrowing of a single mode[8]. The laser in reference [8] is the same model as that used in our work so we use a similar initial linewidth $\Delta\nu_{FLW_0} \approx 5$ MHz based on the scaling of the laser operating power. Using values for our experiment, such as the back-reflection estimated in Supplementary Note 3, we calculate an achievable FLW ranging from 0.02 to 0.1 Hz

depending on values for Q_d and C. This indicates that our minimum FLW is limited by the TRN floor, as pointed out in reference [9].

Supplementary Note 3: Resonator design and feedback characterization

The resonator device has a Si_3N_4 waveguide consisting of a 15 µm SiO_2 lower cladding, a 40 nm thick and 4 µm wide Si_3N_4 core, and a 6 µm SiO_2 upper cladding (Fig. S2(a)). The outputs of the device contain a taper of 2 µm for improved coupling to both the FPLD and the collection fiber. We measure the resonator quality factor, free-spectral range (FSR), and static resonance tuning with a fiber-coupled, single-frequency 780 nm DBR laser with an isolator between the laser and the device (Fig. S2 (b, c)). The DBR frequency is ramped across a resonance while monitoring the fringes of a fiber MZI for different ring heater powers. By tracking the relative shift in the resonance position we extract a tuning strength of 26.3 MHz/mW (Fig. S2(c, d)).

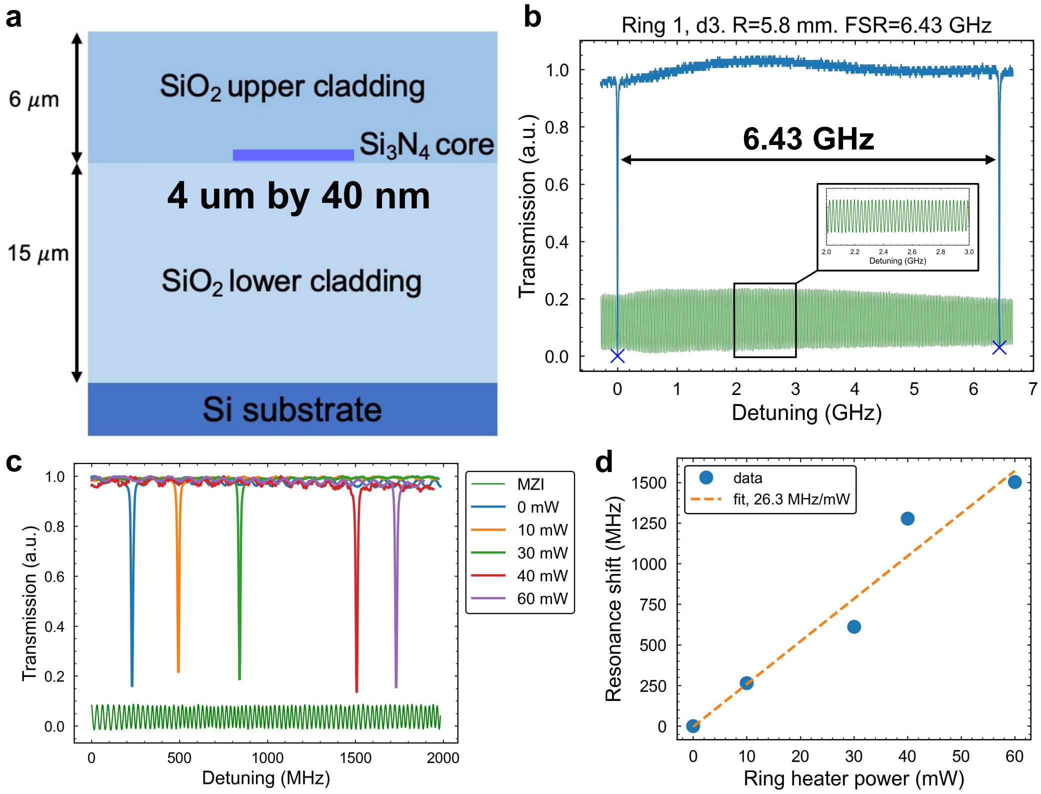

Supplementary Fig. S2. Resonator design and characterization. a) Device waveguide cross-section. b) Free-spectral range (FSR) of the resonator. c) Static tuning with ring heater using a single-frequency DBR laser.

The SIL resonator PIC is designed with a directional coupler splitter. To reduce back-reflections from the terminated end of the splitter we use a spiral with bend radii much smaller than the waveguide critical bend radius and decreasing further after a few turns. This ensures that all the power is radiated out of the waveguides before they reach the termination, preventing any reflections from the termination. In our design, the termination spiral has four turns in an area of 300 by 300 um^2.

Resonator back-reflection measurements. The resonant back-reflection used from the strong frequency-selective feedback is resonance-dependent. For an individual resonance, the fit of the

transmission sweep considers a resonance splitting g due to mode coupling between co- and counter-propagating light and the resonator external, internal, and total linewidths γ_{ext}, γ_{int}, and $\gamma = \gamma_{int} + \gamma_{ext}$, respectively[10].

$$T = \left|1 - \frac{\gamma_{ext}(i\Delta\omega + \gamma/2)}{(i\Delta\omega + \gamma/2)^2 + g^2}\right|^2 \tag{3}$$

where $\Delta\omega$ is the resonance detuning. The level of back-reflection Γ_m is related to these values as[9]

$$|\Gamma_m|^2 = \left|\frac{\gamma_{ext}\, g}{(\gamma/2)^2 + g^2}\right|^2 \tag{4}$$

For the data in Fig. 1c of the main text these values extracted from the fit are show in Fig. S3a below. The total level of back-reflection which considers the resonator and the on-chip splitter is $|\Gamma_m|^2 \approx 0.04$. We also quantified the level of the back-reflection feedback for different resonances by measuring the back-reflected signal while sweeping a single-frequency tunable laser across several resonances over a range 779 to 781 nm using the test setup shown in Fig. S3b. We monitored the outputs at the ring transmission bus and the fiber circulator reflection as shown in the schematic below, calibrating for fiber to PIC coupling loss. This gives us an estimate of the power going into the ring and splitter P_{input} and the back-reflected power P_{refl} in the dashed-line boxed region in the diagram.

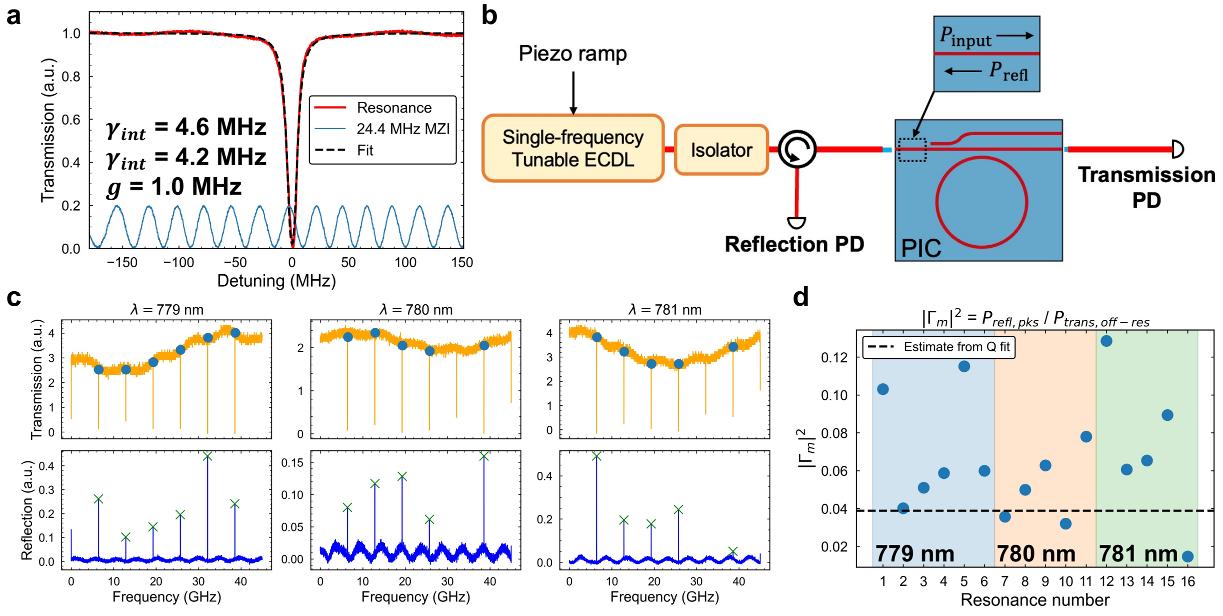

Supplementary Fig. S3. Back-reflection feedback characterization. a) Transmission sweep for a single resonance to extract resonator linewidths and resonance splitting. b) Schematic for measuring the back-reflection. c) Sweep over several resonances for different center wavelengths. d) Calculated back-reflection fraction for different resonances.

The level of back-reflection is given by $|\Gamma_m|^2 = P_{\mathrm{refl}}/P_{\mathrm{input}}$. The result of the frequency sweep is shown in Fig. S3c,d. We estimate $|\Gamma_m|^2$ from the ratio between the reflection peak signal to the off-resonance transmission (blue circles). The strength of the back-reflection is different for each resonance as shown for several center wavelengths of the sweep. The measured back-reflection level is similar to that extracted from the fit of an individual resonance at 780 nm (Fig. 3d, black dashed line). The difference can be attributed to the calibration of fiber-to-chip coupling losses.

Supplementary Note 4: Laser packaging

The coupling between the 90 degree rotated FPLD and the PIC is done using a setup with multi-axis stages and in a packaged version where the FPLD is immobilized with respect to the PIC using UV-cured epoxy. In the main text, the results in Figure 3(c) for laser temperature tuning and one frequency noise trace in Figure 3(d) are measured with the packaged device and all other results are recorded using the stage-coupled device. The image of the packaged device is shown in Figure S4. The PIC devices each contain two SIL ring resonators and one is used at a time by coupling the rotated FPLD at the edge. The PIC is bonded to a circuit board and wire-bonded to control the phase and ring heaters. The entire base-plate is temperature-controlled. In the process of attaching the FPLD, the shrinkage of the UV epoxy led to a significant drop in the laser to PIC coupling efficiency. However, operating the package at an elevated temperature of 50 – 70 deg C improves the coupling efficiency such that at higher laser currents (90 mA) we achieved SIL with 0.7 mW output power. The frequency noise was measured with the MZI OFD method, yielding a $1/\pi$ integral linewidth of 3.1 kHz and a fundamental linewidth of 1.6 Hz (Fig. S4b).

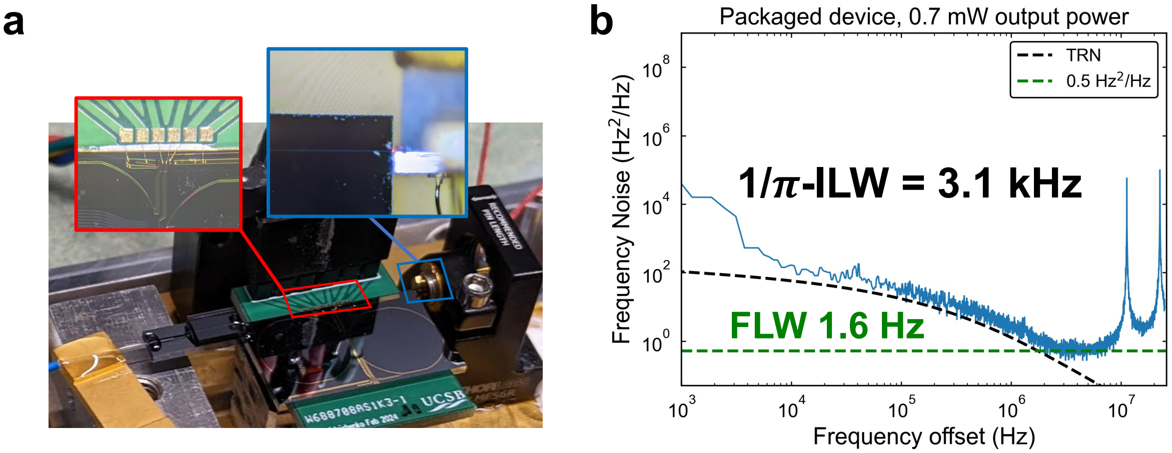

Supplementary Fig. S4. Packaged device. a) Insets: wire-bonding to pads for controlling the phase and ring heaters, zoom in on the coupling region between the FPLD and PIC waveguide input. b) OFD frequency noise measurement for laser current 90 mA and temperature 50 deg C for 792 nm single-mode lasing.

Supplementary Note 5: Atomic systems and laser noise calculations

In this section, we describe the relationship between rubidium-related atomic system performance to the laser frequency noise for gravimeters and high-fidelity neutral atom qubit gates.

Cold atom interferometer gravimeter. In cold atom interferometers based on two-photon transitions, atomic wave packets are split and combined using light pulses of counter-propagating Raman beams. Differences in the phase accumulated in the atomic wavefunctions can be extracted when the atoms are recombined and can be used to extract inertial information such as acceleration due to gravity. The atom interferometer sensitivity described by the interferometer phase variance can be related to the Raman laser frequency noise $S_{\Delta\nu}(f)$ in Hz^2 Hz^{-1} as described by[11,12]

$$\sigma_\phi^2 = \int_{f_x}^{\infty} H(f)^2\, S_{\Delta\nu}(f)\, df \tag{5}$$

where the transfer function that samples the laser frequency noise contribution is given by

$$H(\omega) = 16\pi^2 t_d^2 \sin^2\left(\frac{\omega t_d}{2}\right) \frac{|G(\omega)|^2}{\omega^2} \frac{S_{\Delta\nu}(\omega)}{2\pi} \tag{6}$$

where

$$G(\omega) = \frac{4i\Omega_R}{\omega^2 - \Omega_R^2} \sin\left(\frac{\omega(T + 2\tau_R)}{2}\right) \times \left(\cos\left(\frac{\omega(T + 2\tau_R)}{2}\right) + \frac{\Omega_R}{\omega}\sin\left(\frac{\omega T}{2}\right)\right) \tag{7}$$

is the Fourier transform of the interferometer sensitivity function that relates phase to atomic state transition probability. Here, $\omega = 2\pi f$ is the angular frequency, T is the time interval between Raman pulses, and $t_d = 2L/c$ is the retro-reflector delay for a distance L between the mirror and the atoms. The gravity measurement sensitivity is calculated from the interferometer phase error as

$$\sigma_g = \frac{\sigma_\phi}{g\, k_{\text{eff}} T^2 \sqrt{\Delta f}} \tag{8}$$

where g is the acceleration due to gravity (9.8 m s^{-2}), k_{eff} is the effective wave vector of the two Raman probe lasers, and Δf is the atom interferometer cycle rate or bandwidth. Here, Ω_R is the Rabi frequency of the stimulated Raman transition and τ_R is the time duration of the $\pi/2$ Raman pulses such that $\Omega_R = \pi/(2\tau_R)$. We use the parameters from [12] and set $T = 50$ ms, $\tau_R = 6$ μs, and sensor bandwidth $\Delta f = 4$ Hz, and $L = 46$ cm. The transfer function has a low-pass filtering effect with a corner frequency given by $f_c = \sqrt{3}/(12\tau_R) = 24$ kHz in this example, hence the choice of relevant frequency offset in Table 1 of the main text. Although the gravity sensitivity in many cold atom interferometer gravimeters is limited by vibration noise (200 nm s^{-1} Hz$^{-1/2}$) this analysis shows that lower-noise Raman lasers can improve the atom interferometer noise budget.

High-fidelity neutral atom quantum gates. We use the laser frequency noise to estimate the averaged error of a one-photon transition gate operation based on Equation 78 in reference[13]. Here the error $\mathcal{E} = 1 - \mathcal{F}$ where $\mathcal{F}$ is the gate fidelity. In this calculation we use a Rabi frequency $\Omega_0/2\pi = 1$ MHz and consider a π pulse[13]. The state averaged error is given by

$$\bar{\mathcal{E}} = \frac{8\,\pi^2}{3}\int_{f_x}^{\infty} S_{\Delta\nu}(f)\ H(f) \tag{9}$$

where $S_{\Delta\nu}(f)$ is the driving laser frequency noise (in $\mathrm{Hz}^2\ \mathrm{Hz}^{-1}$). The sensitivity or transfer function for a π pulse is calculated using

$$H(f) = \frac{(\Omega_0^2\ + 4\pi^2 f^2)[1 + \cos(2\pi^2 f/\Omega_0)]}{(\Omega_0^2 - 4\pi^2 f^2)^2} \tag{10}$$

For two-photon and entangled Rydberg excitation gates the requirements on the laser noise are more stringent and a more rigorous derivation can be found in reference [13]. Experiments with ultra-cold rubidium-87 atoms have used several different laser wavelengths for two-photon Rydberg excitation including 480 nm with 780 nm[14], 475 nm with 795 nm[15], and 420 nm with 1013 nm[16]. A specific example of 780 nm laser frequency stabilization in a 480, 780 Rydberg excitation experiment can be found in[17].

References

1. Chauhan, N. *et al.* Visible light photonic integrated Brillouin laser. *Nat. Commun.* **12**, 4685 (2021).

2. Liu, K. *et al.* Integrated photonic molecule Brillouin laser with a high-power sub-100-mHz fundamental linewidth. *Opt. Lett.* **49**, 45–48 (2024).

3. Liu, K. *et al.* Photonic circuits for laser stabilization with integrated ultra-high Q and Brillouin laser resonators. *APL Photonics* **7**, 096104 (2022).

4. Dallyn, J. H. *et al.* Thermal and driven noise in Brillouin lasers. *Phys. Rev. A* **105**, 043506 (2022).

5. Gorodetsky, M. L. & Grudinin, I. S. Fundamental thermal fluctuations in microspheres. *J. Opt. Soc. Am. B* **21**, 697 (2004).

6. Kondratiev, N. M. *et al.* Self-injection locking of a laser diode to a high-Q WGM microresonator. *Opt. Express* **25**, 28167–28178 (2017).

7. Jin, W. *et al.* Hertz-linewidth semiconductor lasers using CMOS-ready ultra-high-Q microresonators. *Nat. Photonics* **15**, 346–353 (2021).

8. Corato-Zanarella, M. *et al.* Widely tunable and narrow-linewidth chip-scale lasers from near-ultraviolet to near-infrared wavelengths. *Nat. Photonics* 1–8 (2022) doi:10.1038/s41566-022-01120-w.

9. Prokoshin, A., Gehl, M., Madaras, S., Chow, W. W. & Wan, Y. Ultra-narrow-linewidth hybrid-integrated self-injection locked laser at 780 nm. *Optica* **11**, 1024 (2024).

10. Puckett, M. W. *et al.* 422 Million intrinsic quality factor planar integrated all-waveguide resonator with sub-MHz linewidth. *Nat. Commun.* **12**, 934 (2021).

11. Zhang, X. *et al.* Compact portable laser system for mobile cold atom gravimeters. *Appl. Opt.* **57**, 6545 (2018).

12. Le Gouët, J. *et al.* Influence of lasers propagation delay on the sensitivity of atom interferometers. *Eur. Phys. J. D* **44**, 419–425 (2007).

13. Jiang, X., Scott, J., Friesen, M. & Saffman, M. Sensitivity of quantum gate fidelity to laser phase and intensity noise. *Phys. Rev. A* **107**, 042611 (2023).

14. Li, R., Qian, J. & Zhang, W. Proposal for practical Rydberg quantum gates using a native two-photon excitation. *Quantum Sci. Technol.* **8**, 035032 (2023).

15. de Léséleuc, S., Barredo, D., Lienhard, V., Browaeys, A. & Lahaye, T. Analysis of imperfections in the coherent optical excitation of single atoms to Rydberg states. *Phys. Rev. A* **97**, 053803 (2018).

16. Levine, H. *et al.* High-Fidelity Control and Entanglement of Rydberg-Atom Qubits. *Phys. Rev. Lett.* **121**, 123603 (2018).

17. Liu, Y.-Y. *et al.* Spectral filtering of dual lasers with a high-finesse length-tunable cavity for rubidium atom Rydberg excitation*. *Chin. Phys. B* **30**, 074203 (2021).
